# Supplementary material for: Mn2+ induced significant improvement and robust stability of radioluminescence in Cs3Cu2I5 for high-performance nuclear battery
Source: Nat Commun. 2021 Jun 23;12:3879. doi: 10.1038/s41467-021-24185-7 (PMC8222237; doi:10.1038/s41467-021-24185-7)
Supplement: Supplementary file 1 — supplementary information [file 41467_2021_24185_MOESM1_ESM.pdf]

Supplementary information

Mn^{2+} Induced Significant Improvement and Robust Stability of Radioluminescence in $Cs_3Cu_2I_5$ for High-performance Nuclear Battery

*Xiaoming Li, Jiaxin Chen, Dandan Yang, Xi Chen, Dongling Geng, Lianfu Jiang, Ye Wu, Cuifang Meng, Haibo Zeng**

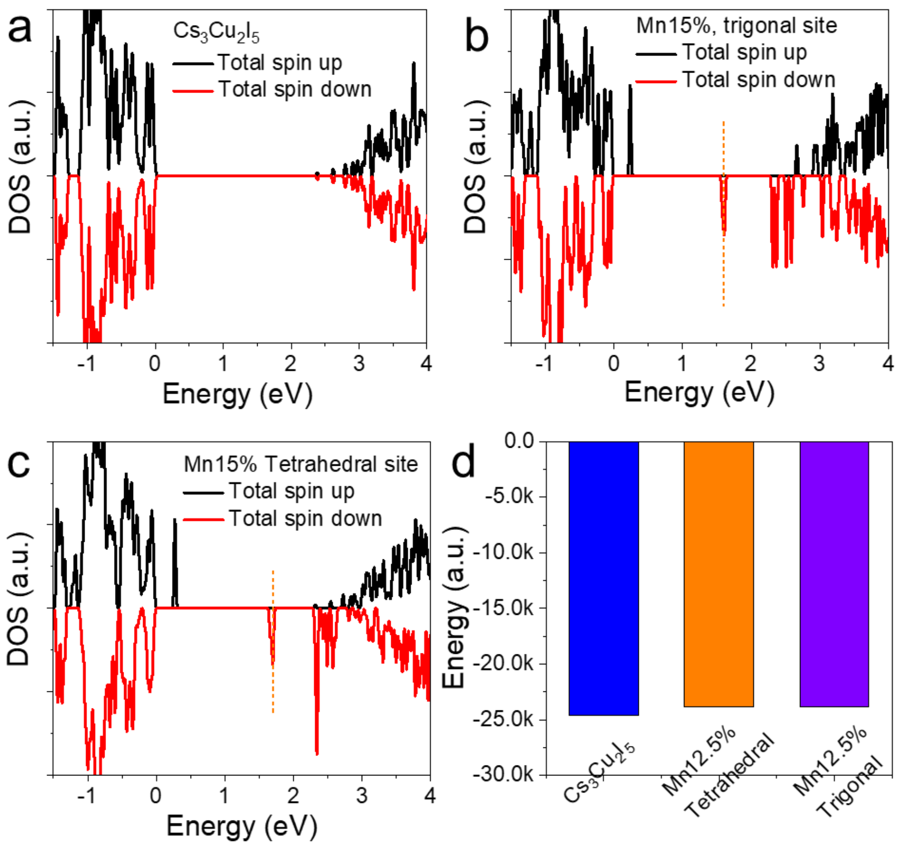

Supplementary Figure 1. Total DOS of (a) intrinsic $Cs_3Cu_2I_5$, (b) Mn3%, and (c) Mn15%. (d) Calculated system energy of $Cs_3Cu_2I_5$ and doped $Cs_3Cu_2I_5$ with different doping sites.

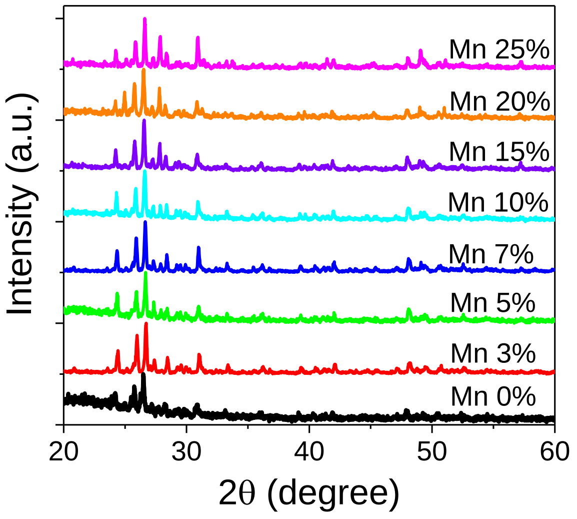

Supplementary Figure 2. XRD patterns with wide diffraction degree range of undoped and doped $Cs_3Cu_2I_5$ powders.

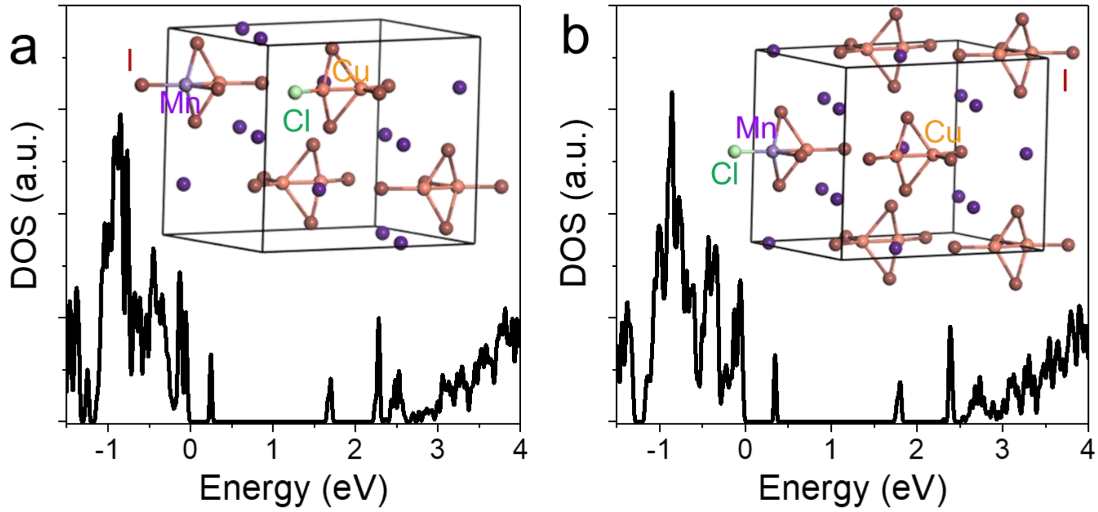

Supplementary Figure 3. Total DOS of $Cs_3Cu_2I_5$: Mn with co-doping of Cl ions at different sites. We can see the incorporation of Cl ions does not change the electronic structures significantly compared to that in Figure S1 except for the change of band gap. The detailed effects of Cl ions in lattice will be investigated in the future, including both the crystal and optical characteristics.

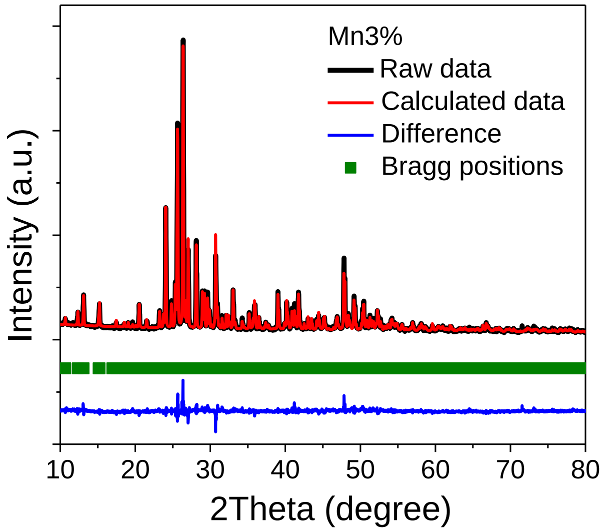

Supplementary Figure 4. Rietveld refinement of Mn3%.

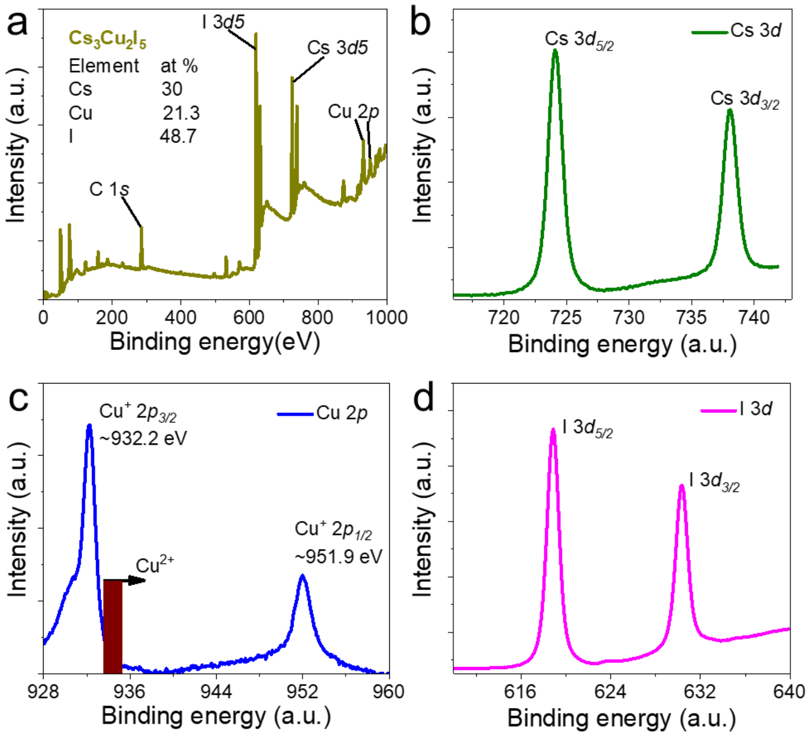

Supplementary Figure 5. XPS results of undoped $Cs_3Cu_2I_5$. (a) XPS survey spectrum of $Cs_3Cu_2I_5$ and refined curves of (b) Cs 3*d*, (c) Cu 2*p*, and (d) I 3*d*.

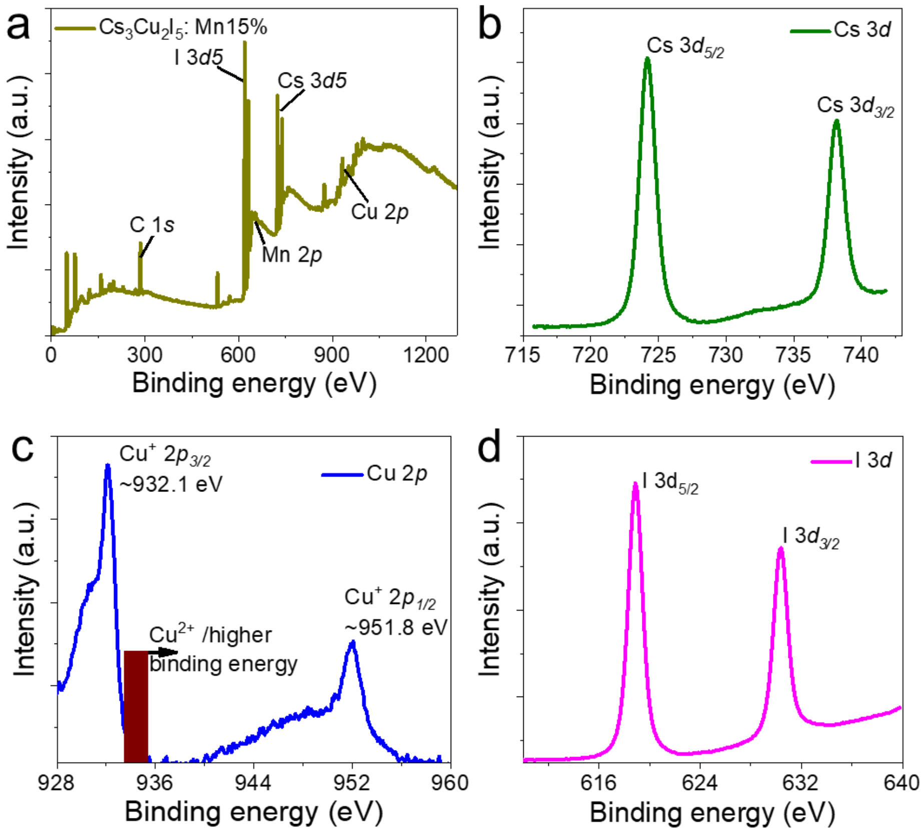

Supplementary Figure 6. XPS results of $Cs_3Cu_2I_5$: Mn15%. (a) XPS survey spectrum and refined curves of (b) Cs 3*d*, (c) Cu 2*p*, and (d) I 3*d*.

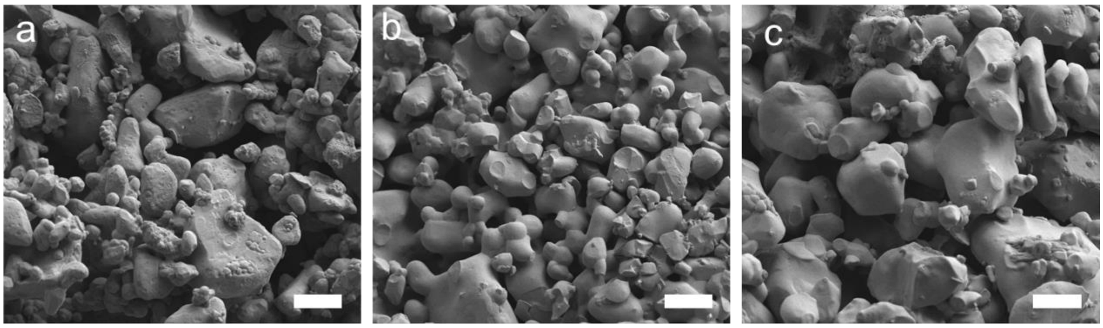

Supplementary Figure 7. SEM images of typical samples with different Mn^{2+} doping concentrations, (a) 0%, (b) 3%, and (c) 15%. The scale bars are 2 μm.

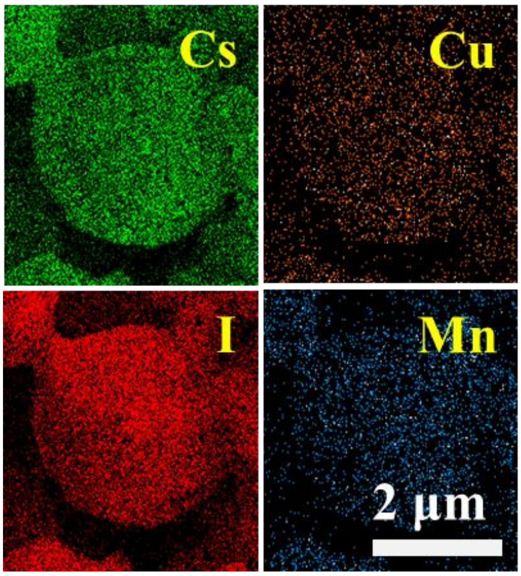

Supplementary Figure 8. Element mapping images of Cs, Cu, I, and Mn for the selected particle.

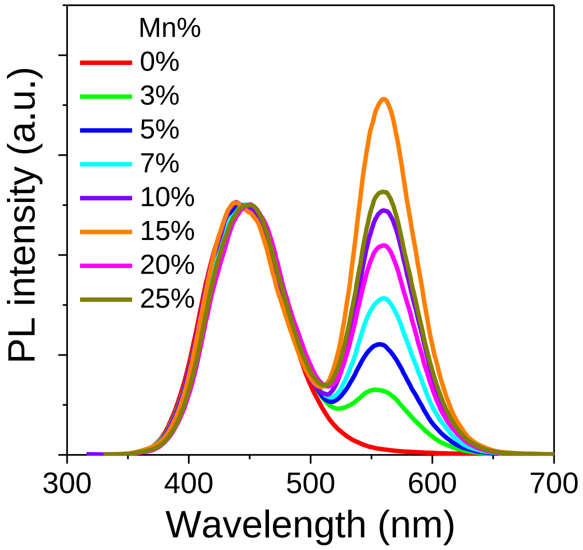

Supplementary Figure 9. Normalized (the intrinsic part) PL spectra of $Cs_3Cu_2I_5$: Mn with different doping concentrations.

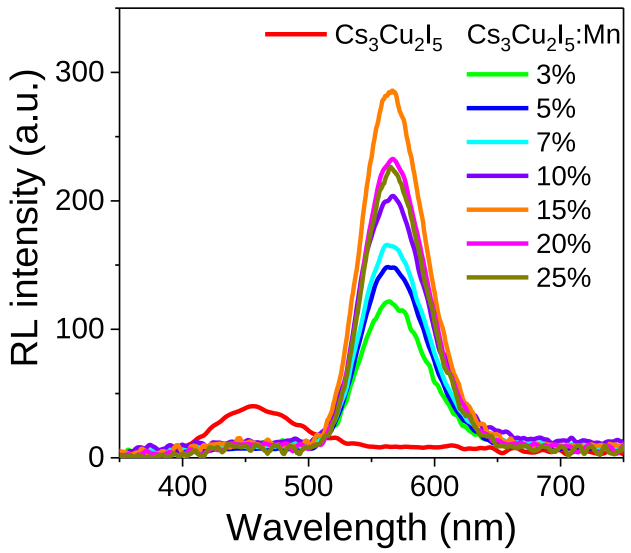

Supplementary Figure 10. RL spectra of $Cs_3Cu_2I_5$: Mn with different doping concentrations.

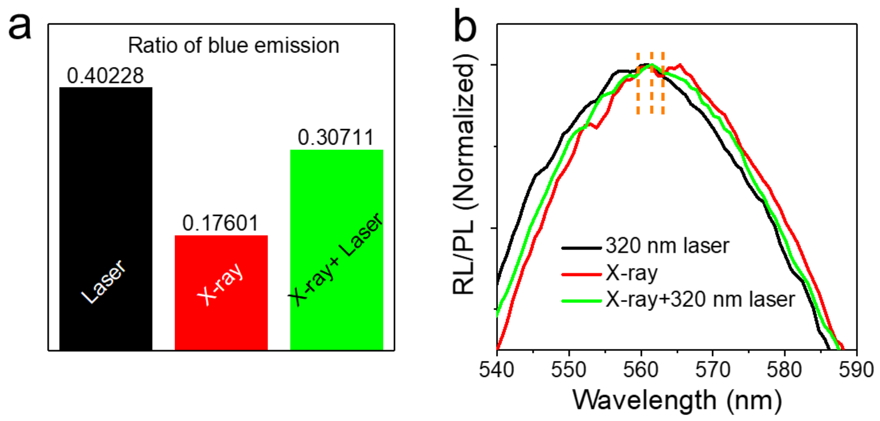

Supplementary Figure 11. (a) The blue emission ratio of curves in Fig. 3a. (b) Enlarged PL and RL spectra of Mn15% film.

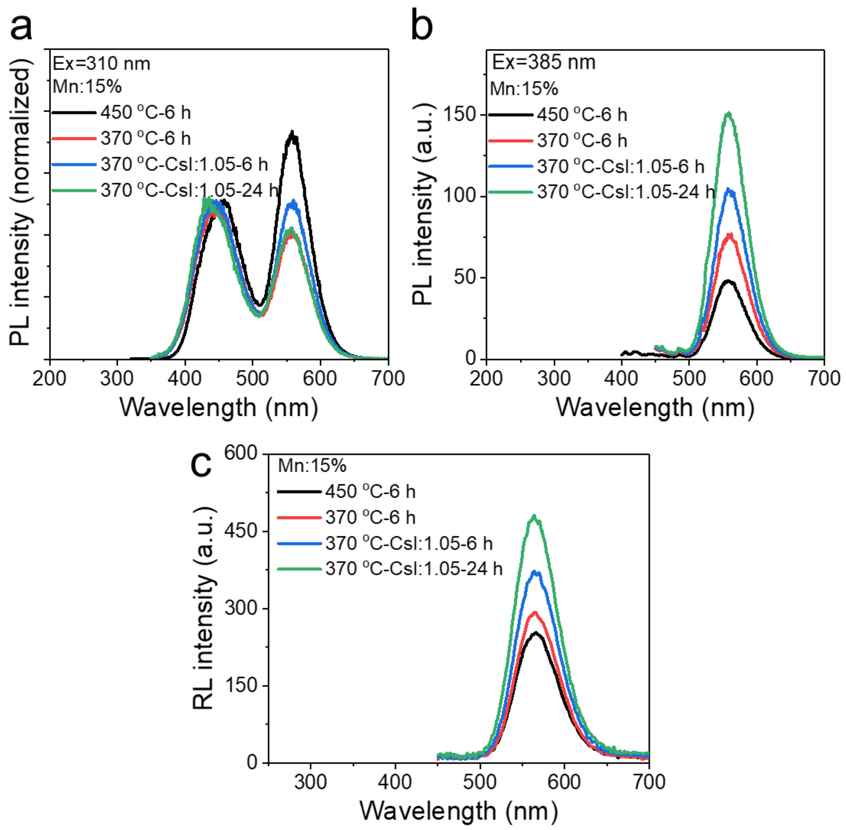

Supplementary Figure 12. Emission spectra of Mn15% with various reaction parameters under different excitations of (a) 310 nm, (b) 385 nm, and (c) X-ray, respectively.

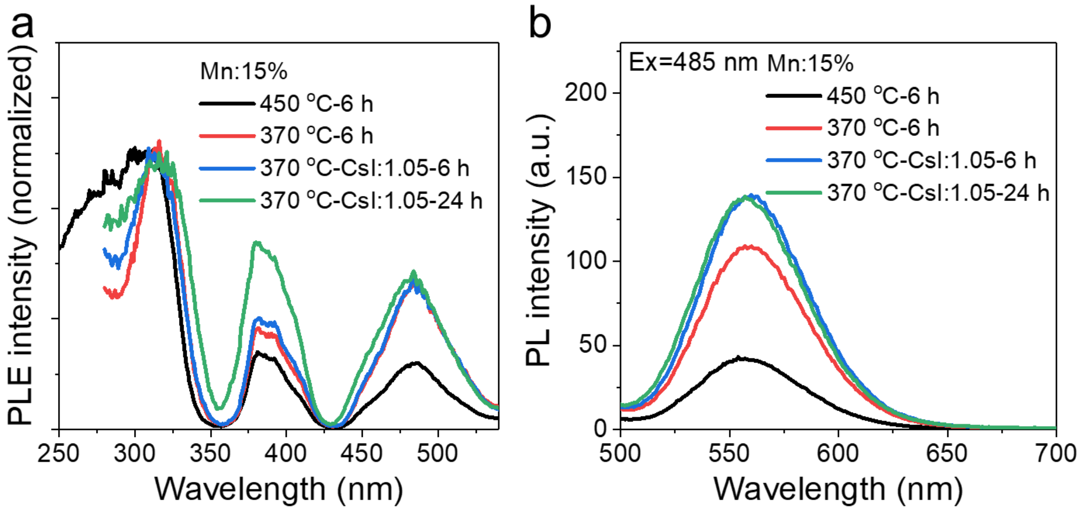

Supplementary Figure 13. (a) PLE spectra of samples with different preparation parameters monitored at 556 nm. Emission spectra of Mn15% with various reaction parameters under excitation of 485 nm light.

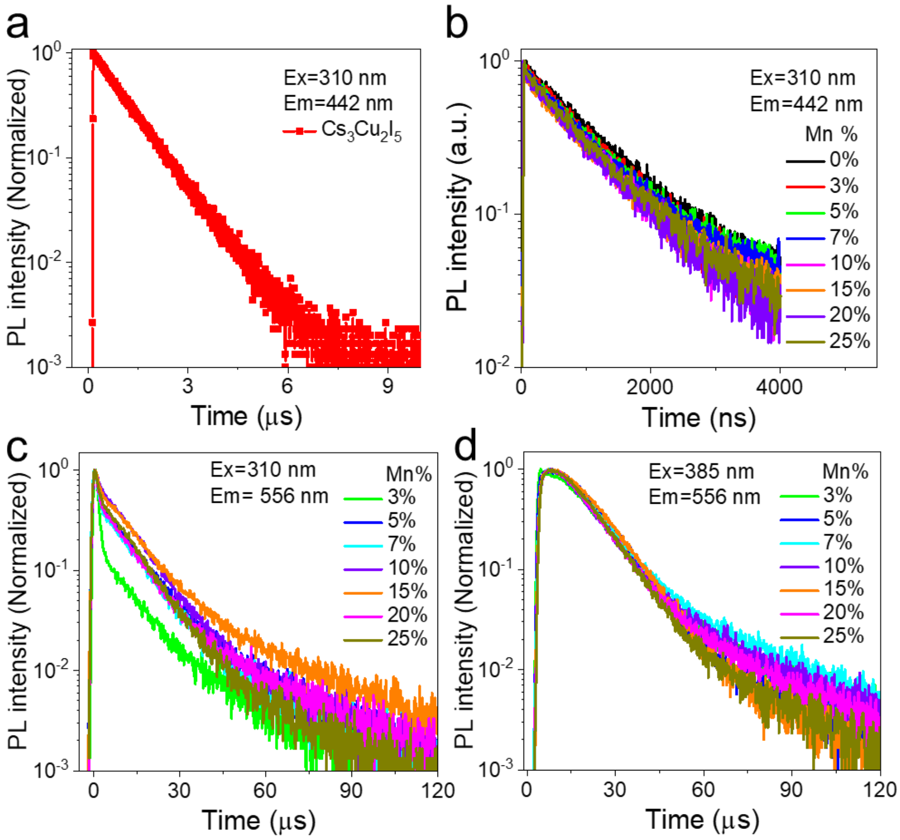

Supplementary Figure 14. PL decay curves of (a) $Cs_3Cu_2I_5$ and (b) $Cs_3Cu_2I_5$: Mn with monitor wavelengths of 442 nm under 310 nm pulse light excitation. Decay curves of $Cs_3Cu_2I_5$: Mn under the excitation of (c) 310 and (d) 385 nm, respectively.

Generally, Mn^{2+} related emission exhibits long PL lifetime to a magnitude of millisecond.[1] Herein, not only the intrinsic $Cs_3Cu_2I_5$ shows a fast decay of 0.96 μs (Fig. S14a) but the Mn^{2+} doped powders also exhibit short average PL lifetime of around ten μs under both indirect and direct excitations (Fig. S14c and d). Such a short luminescence lifetime is adequate for many applications.[2] The fast PL decay can be assigned to strong spin-orbit coupling resulted from heavier halide.[3] All the intrinsic blue luminescence show short decay time less than 1 μs (Fig. S14b). It has been demonstrated that the formation of STEs from free excitons occurs on ps time scales, while the transfer time of exciton to Mn^{2+} is around hundreds of ps. Therefore, the fast and slow components may be related to STE-Mn^{2+} and free-exciton-Mn^{2+} emissions, respectively. First, let us focus on the STE emission. All the samples show a lifetime of

~1 μs with small variation. This value is consistent with previous reported results. With the increase of doping concentration, the decay becomes faster, which can be assigned to the fast energy transfer from STE to Mn^{2+} levels.

For the Mn^{2+} emission, at low doping concentration, STE-Mn^{2+} dominates, resulting in short lifetime. With the increase of doping concentration, free-exciton-Mn^{2+} emission dominates and then leads to longer lifetime. In spite of the dominating role at different doping concentrations, these to emissions exhibit stable decay behavior. Specifically, samples with nominal concentration larger than 15% show similar lifetime and component, indicating the saturation. This is also consistent with other characterizations. Direct excited Mn^{2+} emission possess similar lifetime-scale, this agrees well with previous reports. The detailed lifetime information can be found in Table S3 and S4.

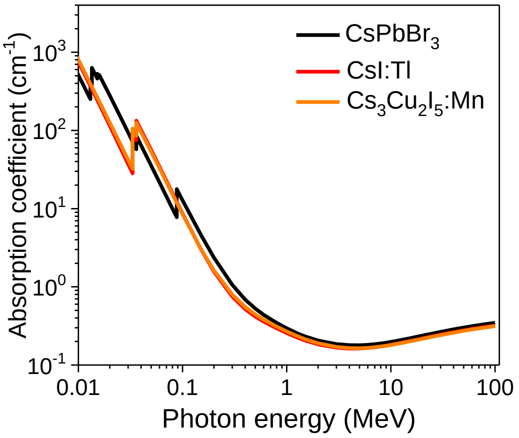

Supplementary Figure 15. Absorption coefficient of different materials as a function of X-ray photon energy.

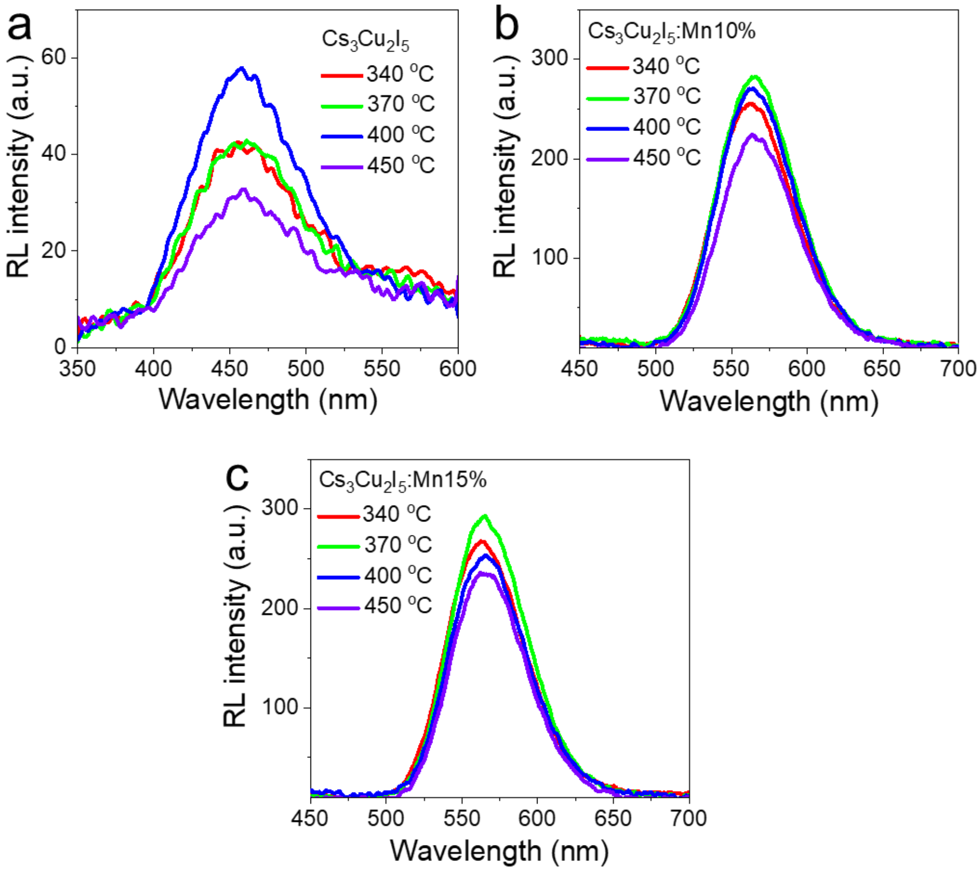

Supplementary Figure 16. RL curves of (a) $Cs_3Cu_2I_5$, (b) Mn10%, and (c) Mn15% prepared under different reaction temperatures.

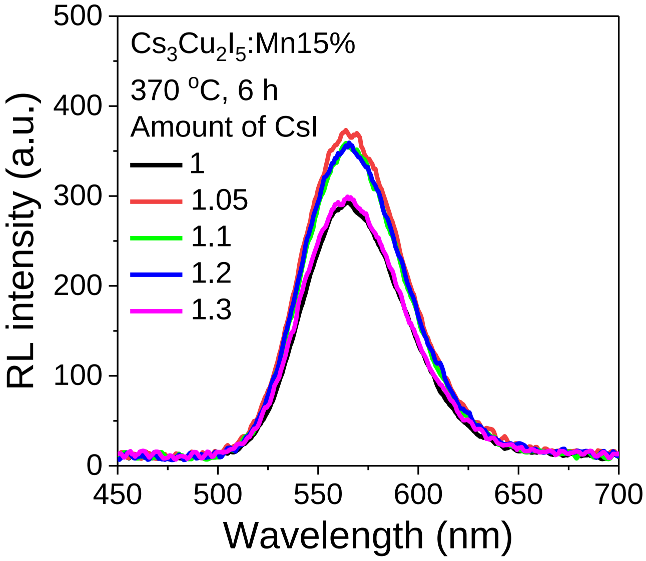

Supplementary Figure 17. RL curves of Mn15% with different CsI mole ratio. All other reaction parameters were kept the same.

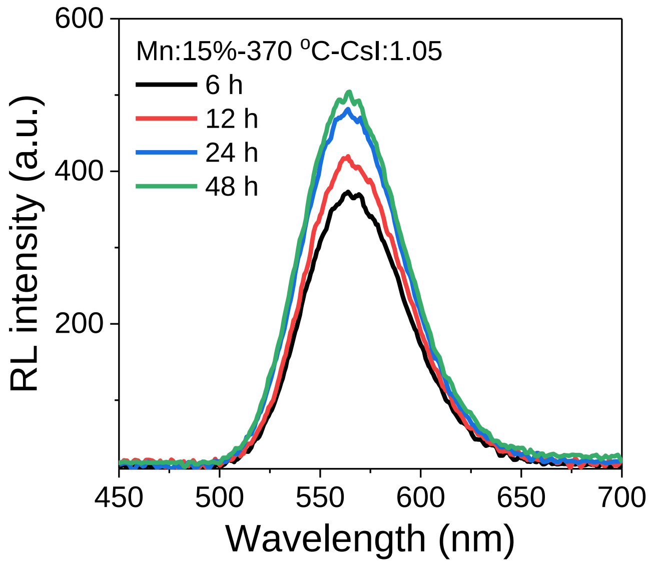

Supplementary Figure 18. RL spectra of Mn15%-CsI 1.05 with different reaction time at 370 °C.

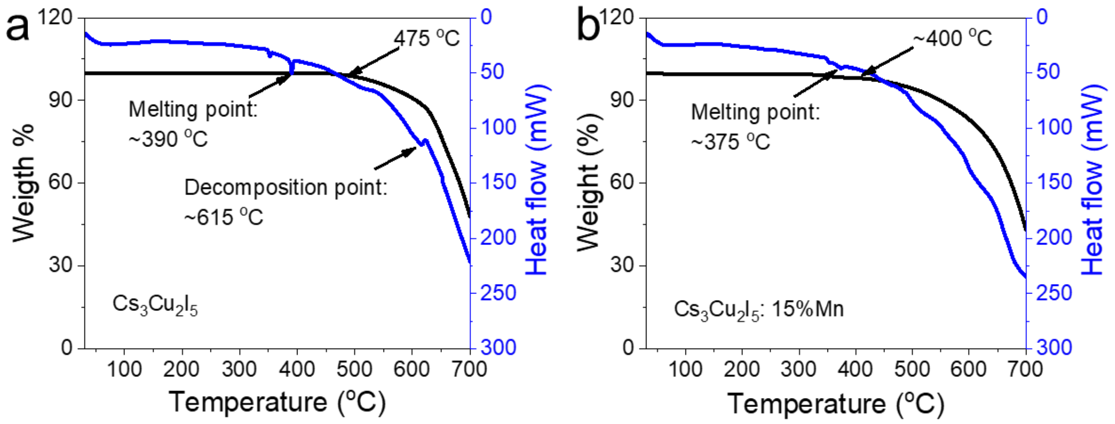

Supplementary Figure 19. Thermal gravimetric analysis (black) and differential scanning calorimetry (blue) TGA/DSC plots for (a) $Cs_3Cu_2I_5$ and (b) Mn15%.

We can see that doping results in decreased melting point. Additionally, the optimal reaction temperatures are related to melting points that the best RL performance was achieved at 400 °C for $Cs_3Cu_2I_5$ while the highest RL intensity was obtained at 370 °C for Mn15%. In fact, the reaction parameters need more investigations for better performance, which are still under research.

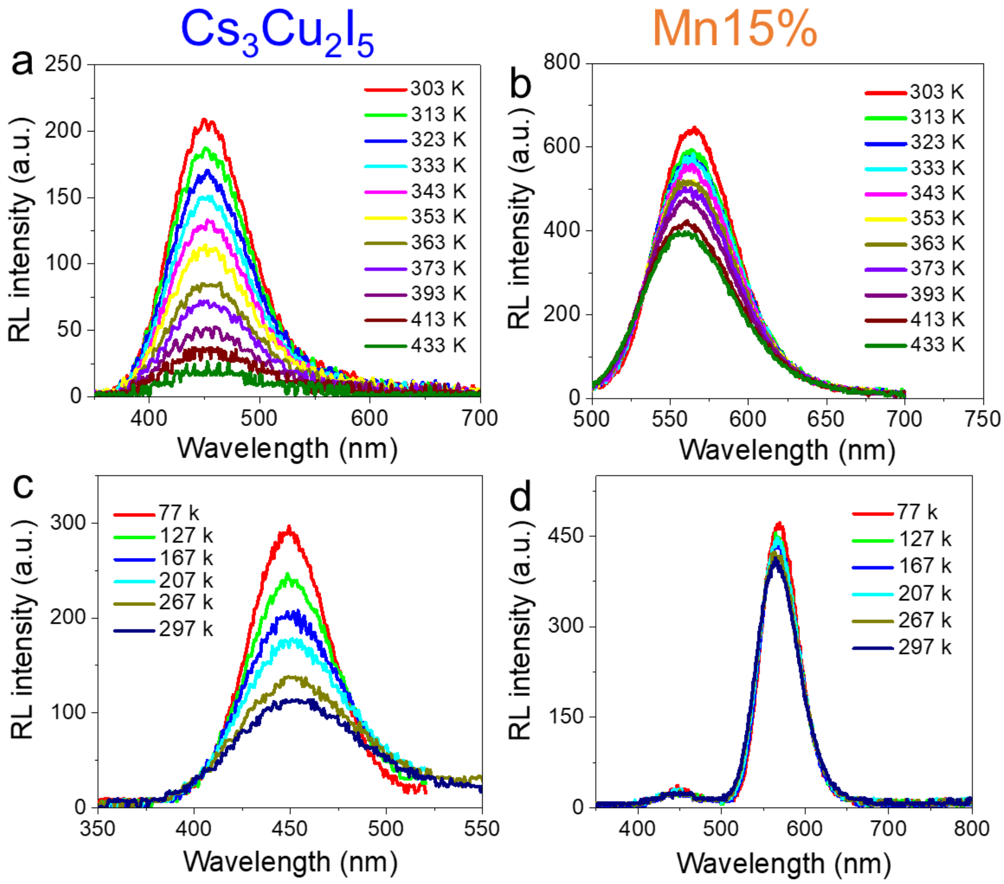

Supplementary Figure 20. RL spectra of (a) $Cs_3Cu_2I_5$ and (b) Mn15% at elevated temperatures. RL spectra of (c) intrinsic and (d) doped $Cs_3Cu_2I_5$ at low temperature. Obviously, without doping, the RL intensity increases upon the decrease of temperature, while the RL intensity almost keep the same.

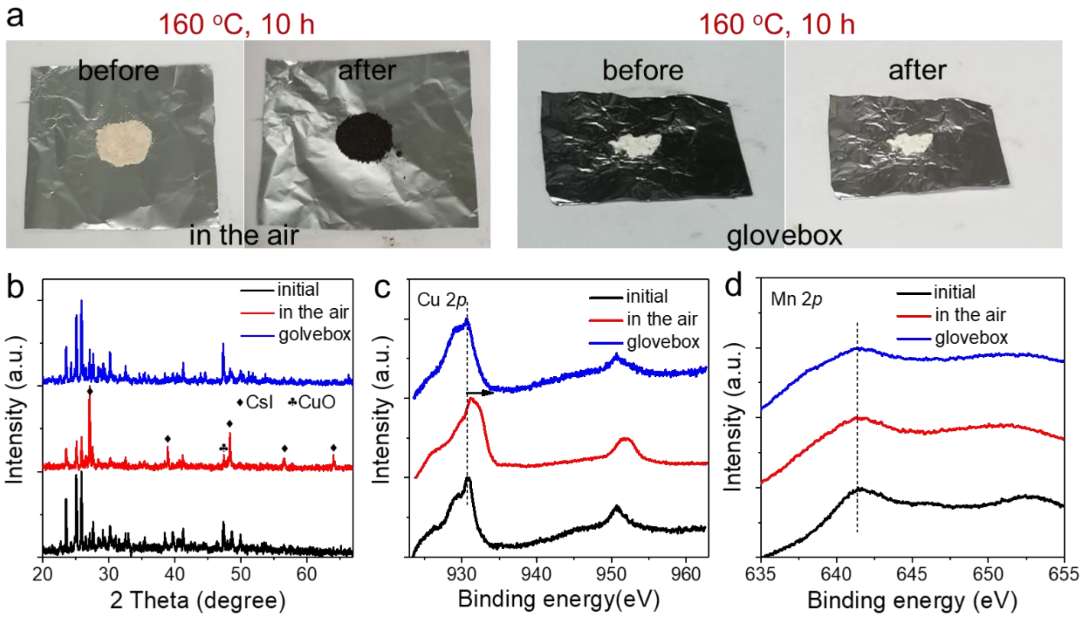

Supplementary Figure 21. (a) Pictures of samples treated in the air or in a glovebox at 160 °C for 10 h. (b) XRD patterns, (c) Cu 2*p*, and (d) Mn 2*p* XPS curves after different treatments.

To further understand the deterioration mechanism against heating treatment in the air, we carried out an extreme experiment to treating the sample (Mn15%) at 160 °C for 10 h both in the air and in the glovebox (argon). As shown in Fig. S22a, when the powders are treated in the air, they become black after 10 h, indicating the possible decomposition, phase transition, or formation of impurities. On the contrary, the powders treated in the glovebox keep the same after 10 h. This means oxygen or moisture is harmful to this material, especially at such a high temperature. Since water will evaporate at high temperature, oxygen is more destructive to the powders considering the color change.

To understand what happens during the treatment in the air, XRD and XPS measurements are carried out. We can see the XRD pattern of the sample treated in the glovebox almost keeps the same expect for the improved crystallinity (Figure R6b), which is consistent with the picture. However, some diffraction peaks related to impurities (CsI, CuO) emerge for the sample treated in the air, indicating the reaction of the material with oxygen and decomposition. Therefore, this is the main reason for the degradation after being heated at high temperature under ambient condition. In spite

of the material degradation, there is still a large content of $Cs_3Cu_2I_5$: Mn from the XRD pattern, contributing to RL continuously. This indicates the oxidation and decomposition happen first on the surface.

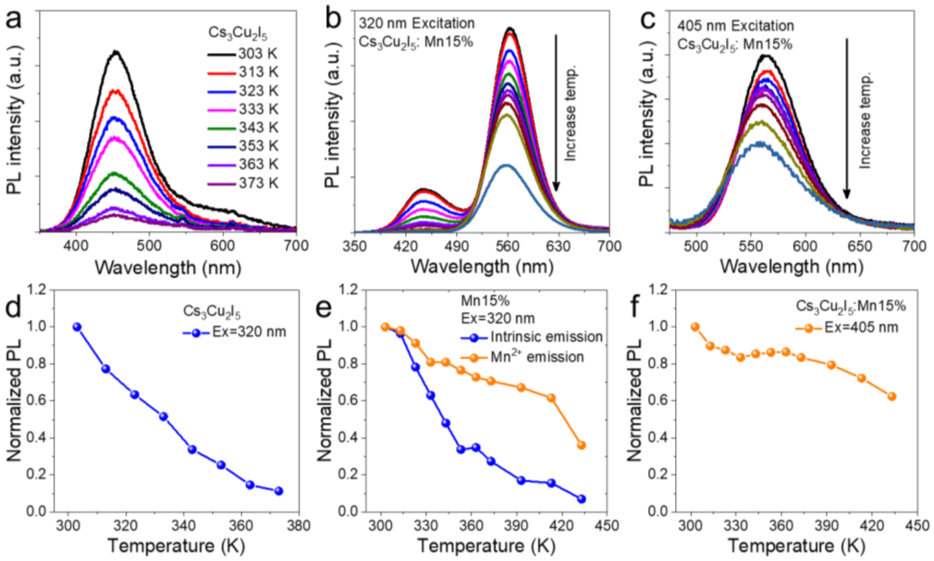

Supplementary Figure 22. Temperature dependent PL spectra of (a) $Cs_3Cu_2I_5$, (b) Mn15% with 320 nm laser excitation, and (c) Mn15% with 405 nm laser excitation. (d-f) Corresponding PL intensity variation of above three samples versus temperature.

Both samples exhibit PL intensity deterioration with the rise of temperature but obvious difference can be observed. For pure $Cs_3Cu_2I_5$, the intensity decreased quickly and only ~10% of its initial value maintained at 373 K. When exciting Mn15% with 320 nm laser light, the intrinsic and Mn^{2+} emission intensities decreased simultaneously but the ET induced yellow emission exhibits a much lower rate. Fig. S20c shows the temperature-dependent PL spectra under the direct excitation of Mn^{2+}. The intensity first decreased with the increase of temperature, which then exhibited a stable intensity in the range of 333 to 363 K, and around 60% of its initial intensity was reserved at 433 K. ET induced and direct Mn^{2+} emissions possess similar thermal stability. Besides, it seems direct excitation leads to better stability, which is consistent with other Mn^{2+} related halides.[4] Such robust resistance to temperature can be assigned to the contribution of thermo-assisted exciton ET and high activation energy of thermal quenching,[2, 5] indicating their potential applications in many fields.

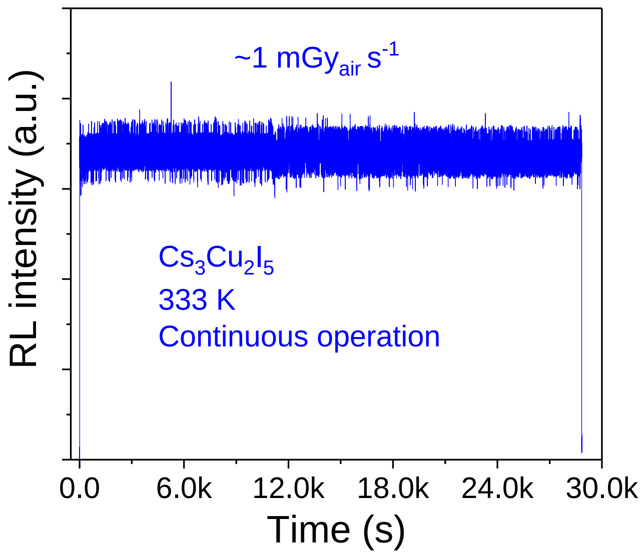

Supplementary Figure 23. RL stability of $Cs_3Cu_2I_5$ against long-term operation at high temperature (333 K).

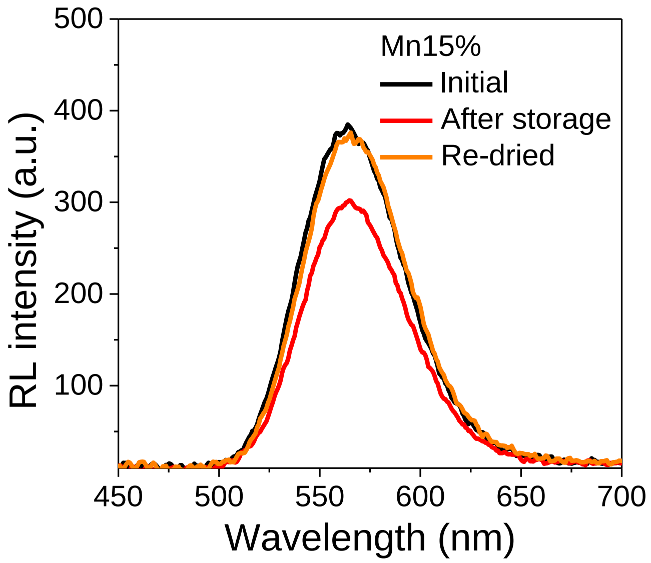

Supplementary Figure 24. RL stabilities of Mn15% after long-term storage without purification.

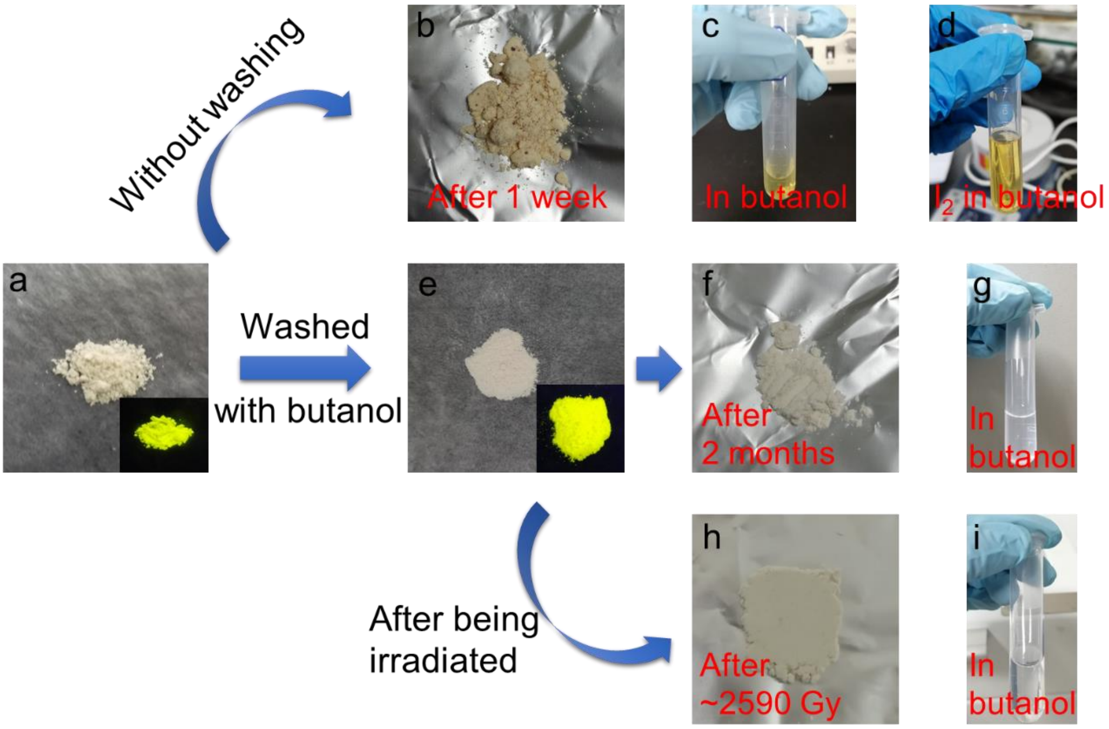

Supplementary Figure 25. Illustration of the deterioration phenomena (a-d), improved stability after purification (e-g), and improved irradiation hardness of the sample (h and i).

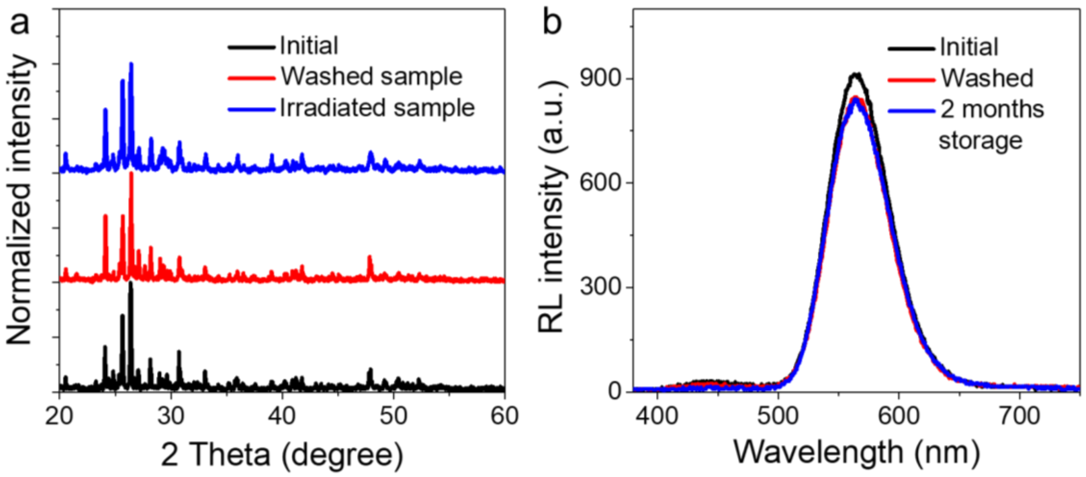

Supplementary Figure 26. (a) XRD patterns of as-prepared, washed and irradiated Mn 15% powder. (b) RL spectra of corresponding samples.

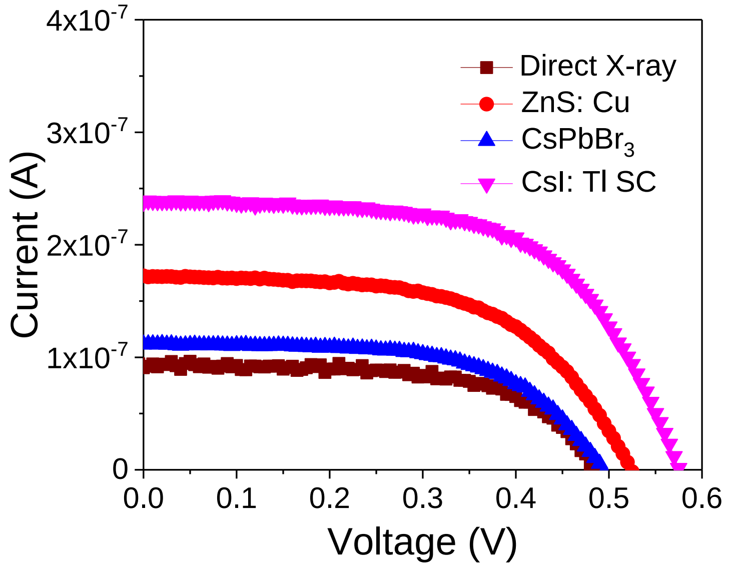

Supplementary Figure 27. I-V curves of NBs with different scintillators.

We can see that $CsPbBr_3$ QDs shows an output improvement of 26.2%, which is comparable with previous report. In fact, $CsPbBr_3$ QDs possess low light yield due to the severe self-absorption effect. ZnS: Cu and CsI: Tl are reported to possess higher light yield, which endow the devices with higher output performance, and improvement of 106% and 214% are achieved, respectively. These results are consistent with the high light yield and long emission wavelength of $Cs_3Cu_2I_5$: Mn.

Supplementary Table S1. Main parameters of processing, refinement, and fractional atomic coordinates of the Mn3% and Mn15% samples.

Formula	$Cs_3Cu_2I_5:Mn^{2+}$(3%)	$Cs_3Cu_2I_5:Mn^{2+}$(15%)
Space group	Pbnm (62) orthorhombic	
Cell parameters	a=14.3462<br>b=10.1589<br>c=11.6369<br>v=1696.00	a=14.3573<br>b=10.1613<br>c=11.6445<br>v=1698.80
Reliability factors	Rp=5.55<br>Rwp=7.03<br>χ^2=2.86	Rp=3.46<br>Rwp=5.21<br>χ^2=4.19

$Cs_3Cu_2I_5:Mn^{2+}$(3%)				
Atoms	x	y	z	occupancy
I2	0.79251	0.84335	0.75000	0.57068
Cs2	0.67741	1.05330	0.98935	1
Cu1	0.63139	0.75634	0.75000	0.49155
Cs1	0.44929	0.40016	0.75000	0.52933
I1	0.55315	0.69025	0.56129	1
I4	0.28017	0.70106	0.75000	0.55978
Cu2	0.45207	0.79094	0.75000	0.49324
I3	0.48786	1.03319	0.75000	0.56410
Mn1	0.63139	0.75634	0.75000	0.00845
Mn2	0.45207	0.79094	0.75000	0.00676

$Cs_3Cu_2I_5:Mn^{2+}$(15%)				
Atoms	x	y	z	occupancy
I2	0.79518	0.83911	0.75000	0.62482
Cs2	0.67969	1.06108	0.99148	1
Cu2	0.62996	0.76144	0.75000	0.49715
Cs1	0.45327	0.39830	0.75000	0.49395
I1	0.55100	0.69088	0.55929	1
I4	0.27805	0.70018	0.75000	0.51262
Cu1	0.44708	0.79052	0.75000	0.41738
I3	0.48823	1.02854	0.75000	0.60761
Mn1	0.44708	0.79052	0.75000	0.08262
Mn2	0.62996	0.76144	0.75000	0.00285

Supplementary Table S2. Detailed QY results of samples with different Mn^{2+} doping concentrations under both indirect and direct excitations.

Samples	QY/310 nm (%)	QY/385 nm (%)
$Cs_3Cu_2I_5$	38.3	--
Mn3%	10.6	2.7
Mn5%	11.6	2.8
Mn7%	16.5	3.4
Mn10%	18.1	8.7
Mn15%	23.1	10.3
Mn20%	12.9	4.9
Mn25%	11.7	5.8
Mn15%-370 ºC	23.3	14.6
Mn15%-370 ºC/1.05/6 h	28.1	18.4
Mn15%-370 ºC /1.05/24 h	29.9	22.7

Supplementary Table S3. PL decay of samples with different Mn^{2+} doping concentrations under indirect (310 nm) excitations with a monitor peak of 442 nm.

Samples	Lifetime (ns)
$Cs_3Cu_2I_5$	960
Mn3%	904
Mn5%	863
Mn7%	852
Mn10%	850
Mn15%	833
Mn20%	857
Mn25%	860

Supplementary Table S4. PL decay of samples with different Mn^{2+} doping concentrations under indirect (310 nm) or direct (385 nm) excitations with a monitor peak of 556 nm.

**Samples**	**Lifetime/310 nm (μs)**	**$\tau_{average}$ (μs)**	**Lifetime /385 nm (μs)**
$Cs_3Cu_2I_5$	--	--	--
Mn3%	1.03/46.1%, 13.48/53.9%	7.74	16.84
Mn5%	1.27/13.9%, 12.08/86.1%	10.58	15.01
Mn7%	1.10/11.9%, 12.53/88.1%	11.14	14.95
Mn10%	0.99/6.2%, 12.42/93.8%	11.71	16.09
Mn15%	0.95/4.6%, 11.40/95.4%	10.92	13.02
Mn20%	1.08/12.2%, 11.44/87.8%	10.17	13.25
Mn25%	1.27/9.4%, 11.38/90.6%	10.42	13.43

Supplementary Table S5. Device performance comparison of nuclear batteries with different scintillators.

Scintillator	Emission source	Current (μA)	Voltage (kV)	P_{max} without Scintillator	P_{max} (μW)	Increased percentage	Ref.
**$Cs_3Cu_2I_5$:Mn film**	**X-ray**	**80**	**50**	**0.027**	**0.091**	**237%**	**This work**
$CsPbBr_3$ Quantum Dot Films	X-ray	1000	50	1.19	1.34	12.6%	6
$CsPbBr_3$ QD/PPO solution	X-ray	800	60	0.3	0.8	133%	7
$CsPbBr_3$ QD/PPO film	X-ray	800	60	--	1.38×10^{-6}	--	8
ZnS: Cu	X-ray	800	30	0.527	0.797	51.2%	9
Ultima Gold	X-ray	500	25	--	0.00478	--	10
ZnS: Cu	β -ray	--	--	--	2.1	--	11

Reference

1. Xie, R.-J., Hirosaki, N., Liu, X.-J., Takeda, T. & Li, H.-L. Crystal structure and photoluminescence of Mn2+–Mg2+ codoped gamma aluminum oxynitride (γ-AlON): A promising green phosphor for white light-emitting diodes. *Applied Physics Letters* **92**, 201905 (2008).
2. Du, P., Luo, L. & Cheng, W. Neoteric Mn2+-activated Cs3Cu2I5 dazzling yellow-emitting phosphors for white-LED. *Journal of the American Ceramic Society* **103**, 1149-1155 (2020).
3. Morad, V. et al. Manganese(II) in Tetrahedral Halide Environment: Factors Governing Bright Green Luminescence. *Chemistry of Materials* **31**, 10161-10169 (2019).
4. Su, B., Molokeev, M.S. & Xia, Z. Mn2+-Based narrow-band green-emitting Cs3MnBr5 phosphor and the performance optimization by Zn2+ alloying. *Journal of Materials Chemistry C* **7**, 11220-11226 (2019).
5. Ni, J. et al. Novel luminescent properties and thermal stability of non-rare-earth Ca-α-sialon:Mn2+ phosphor. *Journal of Luminescence* **202**, 514-522 (2018).
6. Xu, Z. et al. CsPbBr3 Quantum Dot Films with High Luminescence Efficiency and Irradiation Stability for Radioluminescent Nuclear Battery Application. *ACS applied materials & interfaces* **11**, 14191-14199 (2019).
7. Chen, W. et al. Novel radioluminescent nuclear battery: Spectral regulation of perovskite quantum dots. *International Journal of Energy Research* **42**, 2507-2517 (2018).
8. Chen, W. et al. Radioluminescent nuclear battery containing CsPbBr3 quantum dots: Application of a novel wave - shifting agent. *International Journal of Energy Research* **43**, 4520-

4533 (2019).

9. Zhang, Z. et al. Use the Indirect Energy Conversion of the Phosphor Layer to Improve the Performance of Nuclear Batteries. *Energy Technology* **6**, 1959-1965 (2018).

10. Zhang, Z. et al. Application of liquid scintillators as energy conversion materials in nuclear batteries. *Sensors and Actuators A: Physical* **290**, 162-171 (2019).

11. Jiang, T., Xu, Z., Meng, C., Liu, Y. & Tang, X. In‐Depth Analysis of the Internal Energy Conversion of Nuclear Batteries and Radiation Degradation of Key Materials. *Energy Technology*, 2000667 (2020).
